# DIALS as a toolkit

**DOI:** 10.1002/pro.4224

**Published:** 2021-11-24

**Authors:** Graeme Winter, James Beilsten‐Edmands, Nicholas Devenish, Markus Gerstel, Richard J. Gildea, David McDonagh, Elena Pascal, David G. Waterman, Benjamin H. Williams, Gwyndaf Evans

**Affiliations:** ^1^ Diamond Light Source Ltd, Harwell Science and Innovation Campus Didcot UK; ^2^ STFC Rutherford Appleton Laboratory Didcot UK; ^3^ CCP4, Research Complex at Harwell, Rutherford Appleton Laboratory Didcot UK; ^4^ Rosalind Franklin Institute, Harwell Science and Innovation Campus Didcot UK

**Keywords:** methods development, open source, software, X‐ray crystallography

## Abstract

The DIALS software for the processing of X‐ray diffraction data is presented, with an emphasis on how the suite may be used as a *toolkit* for data processing. The description starts with an overview of the history and intent of the toolkit, usage as an automated system, command‐line use, and ultimately how new tools can be written using the API to perform bespoke analysis. Consideration is also made to the application of DIALS to techniques outside of macromolecular X‐ray crystallography.

## 1 INTRODUCTION

### 1.1 A quick history of DIALS

At the beginning of the DIALS project, we had the intention to provide a suite of tools for structural biologists to analyze data, but designed in such a way so that a motivated researcher could “get their hands dirty” by modifying, extending, or adding new algorithms. From experience of using other data processing packages at the time (e.g., MOSFLM^1^ and XDS^2^) it was felt that a package which more followed the design principles of d*TREK^3^ would most directly assist with meeting this goal, as individual components could be replaced or chained together in different ways.

At the same time, our collaborators at Lawrence Berkeley National Laboratory were looking to extend CCTBX^4^ to include data processing tools with a particular focus on X‐ray free electron lasers.^5^ The structure of CCTBX is built explicitly around a collection of open source toolboxes which together form the foundation of projects such as PHENIX^6^ and OLEX2.^7^ We chose to base our developments on CCTBX as:

- the software was already openly licensed in a manner consistent with our commitments,
- the philosophy of toolboxes was consistent with our intentions,
- many useful algorithms were already in place, for example handling of symmetry,
- the structure of hybrid Python / C++ programming fit well with our needs,
- CCTBX already existed, with a set of language choices and build system, which meant we did not need to decide on these within a new project.

The last of these could appear to be trivial, however, should not be understated; getting any software project moving takes considerable effort, and navigating the choices can take a significant amount of discussion. Inheriting choices from an existing project side‐steps this problem entirely, even if it is necessary to revisit some of the implications once the project matures.

Starting from CCTBX, the initial efforts in DIALS were based around two areas: reproducing calculations performed in MOSFLM and XDS, and defining the underlying data structures to achieve this goal.^8^ The first version of DIALS^9^ represented a functional replacement for MOSFLM, with other existing tools still required to determine the symmetry and scale the data (typically, POINTLESS^10^ and AIMLESS^11^), but introduced 3D profile‐fitting similar to XDS. The next phase of development implemented scaling and point group determination^12^ giving a complete data reduction pipeline within DIALS. Throughout these developments, small tools were introduced and retired around the periphery, and the underlying architecture was modified from time to time, for example, changing reflection file formats. One decade after the project started, DIALS has grown into an international open source collaboration, with 35 contributors to the DIALS repositories.

### 1.2 The idea of DIALS as a toolkit

The idea of DIALS as a toolkit comes from the original design: the authors wanted software which may be applied in new ways, or extended, without a substantial burden. We were also aware that the algorithms could be applied outside macromolecular crystallography, for example, in chemical crystallography, and also outside the arena of rotation crystallography. This can only really be achieved if *all* the software is implemented as a toolkit, as then small components can be modified or replaced without impact on the overall system behavior. However, as with any software development, there are limited resources, so at times our goals may not have been realized as completely as we may have liked. For example, documentation always lags behind development, though we do have a useful set of user tutorials. Overall DIALS does provide a useful starting point for further work, as demonstrated with current efforts to extend DIALS toward processing electron and neutron diffraction data (see Section 5).

### 1.3 Approach of the manuscript

The aim of this manuscript is to present the “toolkit view” of DIALS to the busy structural biologist, not necessarily as a didactic work, but rather to illustrate its potential. The structure chosen is top‐down, starting from the highest level tools, with which a reader may already be familiar, and working toward the lower level features which may be less accessible to the nonexpert.

## 2 AUTOMATED DATA PROCESSING

From the outset, DIALS was built to support existing automated data reduction tools, such as xia2.^13^ It was felt, even then, that interactive data processing should be increasingly reserved for the most complex or challenging cases, with the majority of data processed automatically on behalf of the user. One implication of this was that DIALS favors reliable, automated decision making using a robust set of algorithms over interactive operation requiring users to verify decisions. Most importantly, however, a deliberate choice was made to separate the fundamental algorithms from the “crystallographic decision making,” so that the former resides in the DIALS package, and the latter inside xia2, which is included within the DIALS distribution and also openly licensed.

### 2.1 Basic data processing: xia2

The use of xia2 is well documented elsewhere—in essence it makes the decisions on behalf of the user to process data from X‐ray diffraction images to scaled intensities with no interactive user input, by default using DIALS for most of the key steps. Overall the process is:

- “import” the data for example, read image headers, make choices about the implied structure of the data (MAD, multi‐sweep etc.), then,
- perform spot‐finding, indexing, lattice estimation, refinement and integration on every sweep, then
- scale all data together, by default combining data from common wavelengths into single reflection lists.

There are some slight deviations from this. For example, in “small molecule” mode (xia2.small_molecule) data from all sweeps are indexed simultaneously before being independently refined. In the general case, however, xia2 follows the same workflow for processing with DIALS as before for XDS and MOSFLM. The key point is that anything which can be done automatically with xia2 can be achieved interactively using the DIALS tools.

### 2.2 Usage

In the simplest case, using xia2 is as simple as typing xia2/path/to/data, though usually a few more options will be applied, for example, setting anomalous = true or picking out specific data sets with image=/path/to/data/file_0001.cbf or image=/path/to/data/file_master.h5. In some cases, the user may have insight into the unit cell and symmetry, whereupon the options space_group= and unit_cell= may be used. In the vast majority of cases, this is not necessary, but where, for example, you have multiple sweeps and there are multiple correct but inconsistent cell choices, it may be useful to select one in advance. An example of this is where the cell angles are close to a boundary where the “best” choice for *β* can switch to 180°− *β*, leading to inconsistent indexing between sweeps. If the unit cell is given, the space group must also be given.

Another useful option is:






  xia2 image=/path/to/data/file_master.h5:1:3600






which will only consider images 1 to 3,600 found in file_master.h5—useful in the case where significant radiation damage was found, or the sample moved out of the beam. Multiple copies of this option may be made to include “good bits” of data from a poorly‐centered sample which has moved in and out of the beam. One final variation on this theme is to use “chunking” in the processing of the data with:






                xia2 image=/path/to/data/file_master.h5:1:36000:3600






which will process all 36,000 images in independent blocks of 3,600 images. Ideally, the block size is sufficiently large that the unit cell and other parameters are well determined. One use of this is to make processing of very large data sets more tractable with modest computational resources, but it is more useful in cases where equally sized data sets are recorded from different samples as part of the same acquisition (e.g., [https://zenodo.org/record/1442922] acquired using the ZOO data collection system at SPring8^14^).

This last mode illustrates one of the key features: multi‐crystal processing. By default, multiple data sets are presented to xia2 either in the form of chunks as above, or through multiple data sets being present in a folder, or through multiple image = keywords. They are assumed to come from independent but isomorphous crystals. It may be the case that they are from a single crystal but this is not assumed as the default. Each data set will be indexed and integrated independently, then all data are scaled together. When presented with multiple sweeps, indexing ambiguity (e.g., in polar space groups or as a result of accidental symmetry in unit cell parameters) is resolved by taking the first data set as a reference and reindexing subsequent data sets to match. This works well if the first set is representative and reasonably complete. In the case of a number of highly partial data sets (e.g., the data from SPring8 listed above) this process may be unreliable and the user is advised to consider xia2.multiplex, described below, which makes use of dials.cosym ^15^ to resolve symmetry and indexing ambiguity.

#### 2.2.1 Advanced options

By design, the most common options in xia2 correspond to “sensible defaults,” however there are some cases where some hints may be given to ensure the processing makes the right choices. The most commonly used of these is small_molecule = true, which is aliased to xia2.small_molecule and detailed below. Another very useful option, particularly in cases where a large number of data sets are to be processed, is failover = true which will not stop processing if a data set fails to index or integrate: useful in cases where all data from for example, *in situ* experiments are processed and data quality is variable. If the unit cell has been given, failure to index with this unit cell for a given data set will prevent processing and hence give some crude elimination of nonisomorphous data sets.

In the case where all data are from a single crystal, for example where multiple reorientations are performed with a multiaxis goniometer, it may be desirable to determine a single *UB* matrix for all data sets. This is indicated with multi_sweep_indexing = true (the default for small_molecule = true). This will have the effect of resolving any indexing ambiguity from the outset, but should only be used where the data are genuinely from a single sample recorded without removing and remounting.

#### 2.2.2 Chemical crystallography

From a purely mathematical perspective, X‐ray diffraction from small molecules is no different to that from macromolecules. The differences are however embedded in details—how the data are collected and the greater range of potential space groups, as inversion symmetry may be present. While the latter is most readily recognized, the former has a much greater potential impact and will be considered here.

For chemical crystallography, it is conventional to record a complete data set as a number of “runs” with the sample reoriented and potentially the detector moved between runs.^16^ In some cases, the detector offset may be sufficiently large that there are no low‐resolution regions on the detector, making indexing of the diffraction pattern challenging. The approach taken in xia2.small_molecule is to use a feature of dials.index where spots from all runs may be indexed simultaneously with a single *UB* matrix, which is then subsequently refined independently for each run. This ensures that the data from all sweeps are consistently indexed as well as allowing the data at low resolution to be used to assign indices to data at high resolution. It is obviously necessary to have a reasonably accurate instrument model (i.e., reliable values in image headers) though the process is moderately forgiving as indexing is more tolerant of errors in experimental geometry for the smaller unit cells typical of chemical crystallography.

The question of symmetry, and 230 available space groups as compared with the 65 without inversion centers, is easily resolved: dials.symmetry outputs both the “true” symmetry in the intensities, which includes an inversion due to Friedel's law, and a “macromolecular” space group which is assigned to the output by default. In the small molecule pipeline the “true” symmetry is assigned to the data for scaling, allowing all 230 space groups, though at the cost of potentially assigning the wrong enantiomorphic space group for chiral molecules. Usually, the impact of this assignment is modest, and if unmerged data are used for the subsequent structure determination and refinement, as is common, no impact on the Flack parameters^17^ should be found.

#### 2.2.3 Output

The command‐line output of xia2 is intended to focus on just the core facts—a very brief summary of the key processing steps, followed by the “Table 1” summary for the merged data. A much more comprehensive report is written to xia2.html, which may be viewed in a web browser and includes interactive graphs generated with Plotly. ^1^ This is a richer report, which can also be integrated with automated data processing at facilities so it can be shared online, without the need for the end user to download and manually display files.

**TABLE 1:** Output from dials.symmetry, run on data from a cubic insulin crystal: the distinction between present and absent symmetry operations is clear, as highlighted by the presence or absence of “***”. The number of asterisks indicates the confidence in the presence of the operator

Likelihood	Z‐CC	CC	*N*	Operator
0.906	9.83	0.98	171,140***	1 (0, 0, 0)
0.147	4.33	0.43	338,920	4 (1, 1, 0)
0.149	4.37	0.44	329,464	4 (1, 0, 1)
0.149	4.38	0.44	329,384	4 (0, 1, 1)
0.907	9.74	0.97	329,862 ***	3 (1, 0, 0)
0.907	9.73	0.97	329,794 ***	3 (0, 1, 0)
0.907	9.75	0.97	329,788 ***	3 (0, 0, 1)
0.907	9.74	0.97	329,832 ***	3 (1, 1, 1)
0.906	9.84	0.98	169,216 ***	2 (1, 1, 0)
0.154	4.47	0.45	168,454	2 (−1, 1, 0)
0.907	9.62	0.96	169,212 ***	2 (1, 0, 1)
0.149	4.38	0.44	164,766	2 (−1, 0, 1)
0.907	9.62	0.96	168,726 ***	2 (0, 1, 1)
0.149	4.37	0.44	164,780	2 (0, −1, 1)
0.153	4.45	0.44	170,096	2 (1, 1, 2)
0.149	4.37	0.44	164,764	2 (1, 2, 1)
0.149	4.38	0.44	164,720	2 (2, 1, 1)

In addition to the HTML reports, the scaled data are output in the most commonly used file formats for downstream analysis, namely MTZ and scalepack format (merged and unmerged), additionally with SHELX HKLF4 format in small‐molecule mode.

### 2.3 screen19: Recommendations on data collection with a pixel array detector

**FIGURE 1:**
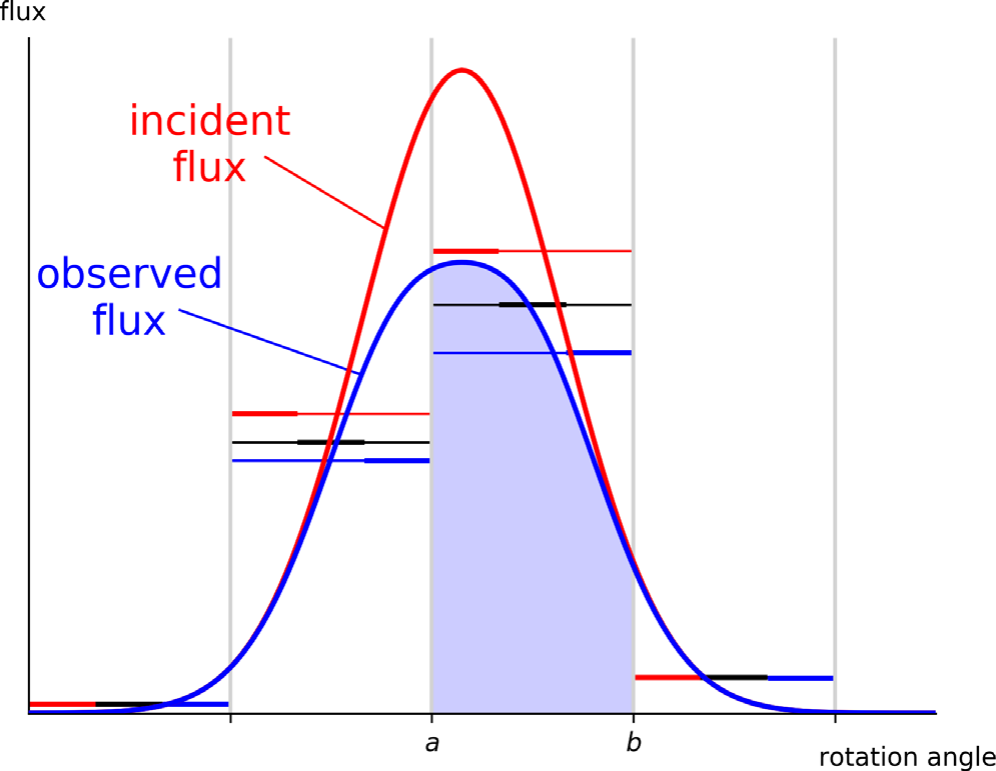
Schematic rocking curve of a single reflection, illustrating the undercounting and count rate correction in a pixel array detector. Horizontal levels indicate the mean true incident flux in each exposure (red), the mean observed flux in each exposure (blue), and the effect on the observed flux of the detector's in‐built count rate correction (black)

With CCD detectors, overloaded pixels were obvious, in that the counts in a pixel would exceed some threshold and so be flagged as invalid. By their very nature, the more important limit for photon‐counting pixel array detectors is on the count *rate* rather than the total counts in a pixel. Of particular importance are pixels that are exposed to large variations in the *instantaneous* count rate, that is, pixels in the vicinity of the peak of the rocking curve of a reflection. Following the measurement of a photon at a receptive pixel, there is a dead period during which the pixel is insensitive to the arrival of further photons.^18^ Naturally, the frequency of these photon pile‐up events increases with incident flux and so this undercounting of photons leads to a nonlinear relationship between flux and count rate. Many pixel array detectors, such as DECTRIS EIGER and PILATUS models, apply a correction to the measured photon count that aims to negate the systematic shortfall.^19^ However, the nonlinearity of the undercounting means that if there is significant variation in instantaneous flux during the exposure, the corrected count will remain an underestimate of the true fluence, as illustrated in Figure 1.

With macromolecular samples, the crystal mosaicity is usually sufficiently large that slicing data finely (e.g., 0.1° rotation per frame) ensures that the variation in instantaneous flux during any single exposure is comparatively small. In chemical crystallography, this may be a long way from the truth, as samples may have very small mosaicity, while also being highly ordered and diffracting very strongly, giving rise to the possibility of significant undercounting.

To address this challenge, a tool named screen19 was developed^2^ which uses DIALS tools to perform spot finding across a small wedge of data, conduct the standard reflection profile modeling in 3D, as would be used in integration (described below) and then compute an estimate of the ratio of the maximum to mean photon flux. If the images are recorded with a rotation significantly finer than the mosaic spread, this ratio will be close to unity. If, however, the mosaic spread is significantly smaller than the rotation width the ratio will be substantially greater than one. This ratio is then used to adjust the maximum trusted pixel value to allow assessment of which pixels may be saturated.

Of course, in addition to the diffraction being stronger than ideal, it is also quite possible that the diffraction may be weak and that a greater exposure may be necessary to achieve the experimental objectives. To this end, screen19 also integrates the data and fits a Wilson distribution^20^ to the resulting intensities, to estimate the extent to which the overall scale of the data needs to be adjusted to reach a set of standard resolutions such as 0.84 Å.^21^ Between the upper limit, saturating the detector, and the lower limit, giving the minimal acceptable *I∕σ*_*I*_, lie a range of sensible experimental parameters to consider.

### 2.4 Automated combination of multiple data sets: xia2.Multiplex

As mentioned above, xia2 may be used to process data from multiple samples to give a complete data set. If the individual data sets are substantial, the symmetry derived from each sweep will be the same, so that the protocol used to resolve any indexing ambiguity, to scale and to merge the data will be successful. If, however, the individual data sets are incomplete, the symmetry determination may be unreliable and depend on which particular symmetry operators are visible in each set. A useful solution to this was developed in dials.cosym (,^15^ and described below), which resolves the crystal symmetry and indexing ambiguity simultaneously. This has been combined with other tools from the DIALS suite to create xia2.multiplex, a tool which takes a number of data sets integrated with DIALS, resolves the symmetry and any indexing ambiguity and then assesses the isomorphism between data sets based on a number of criteria. If requirements for the data set (e.g., overall completeness) are provided, then all hierarchical clusters that achieve those criteria are independently scaled and merged, with summary data available for every cluster. This allows the combination space to be explored effectively while minimizing user interaction time. Once a cluster has been chosen the data are already prepared for downstream analysis.

## 3 COMMAND‐LINE DATA ANALYSIS

The tools described thus far are considered as “high level”; they perform a significant amount of the necessary decision making on the user's behalf. Of course, tools such as xia2 are not actually performing the data processing calculations themselves, instead using, for example, DIALS or XDS to do the actual data processing. As such, any result achieved by xia2 can also be achieved using the lower level DIALS tools, though this involves more interactive work on behalf of the user.

### 3.1 The basic DIALS workflow

**FIGURE 2:**
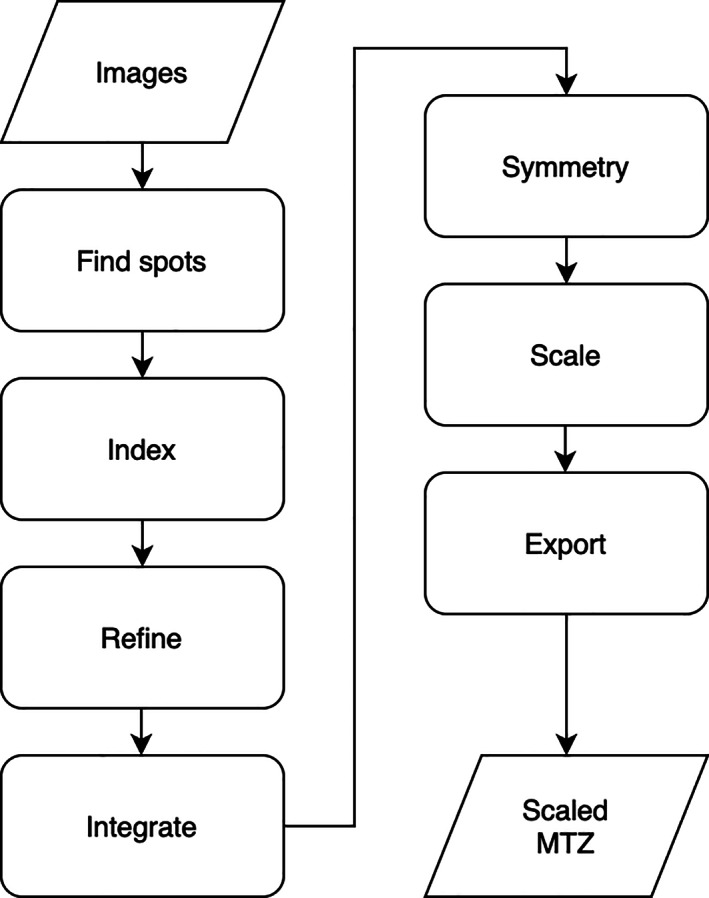
Simple workflow of data processing, which is largely independent of the processing package used

**FIGURE 3:**
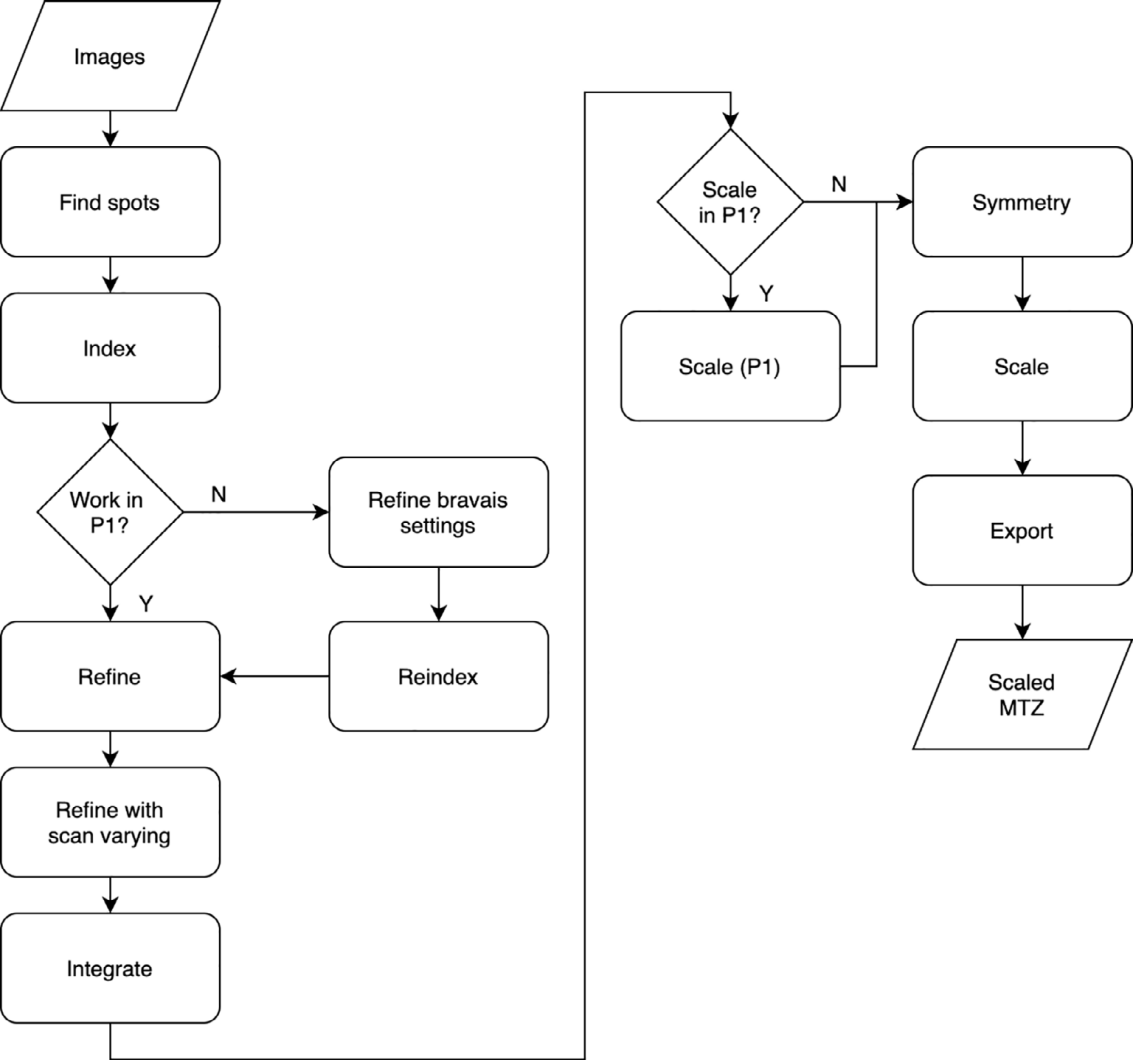
Simple workflow of data processing with DIALS, which deliberately follows the simple workflow shown in Figure 2

The basic workflow using DIALS is necessarily similar to all other integration packages, though of course there are always differences in the detail. The vast majority of the effort behind DIALS has been the development of the main processing pipeline—the steps necessary to index, integrate, and scale X‐ray diffraction data to give useful scaled and merged intensities. To most users these details are hidden behind the scenes, however, the command‐line tools expose a workflow which has much in common with other software such as XDS and d*TREK (Figure 2). With DIALS, the process run by the user typically follows this workflow, starting with importing the data (which reads the image headers and builds a model of the experiment), followed by spot‐finding, indexing, refinement, integration, and so on (Figure 3).

In the simplest case—one complete data set derived from one crystal—the workflow is very straightforward, with only two major decision points: whether to apply the Bravais lattice constraints when processing the data, and whether to scale the data before attempting to derive the correct Patterson symmetry.^3^ For the majority of good quality data sets, these choices will make little difference to the outcome—the correct model is well determined from the outset, and the scaling model varies modestly, thus not affecting the correlation analysis across potential symmetry operations. If, however, the sweep is narrow, or there are large variations in the absolute scale of the data (e.g., from a plate crystal), these choices may be crucial.

**FIGURE 4:**
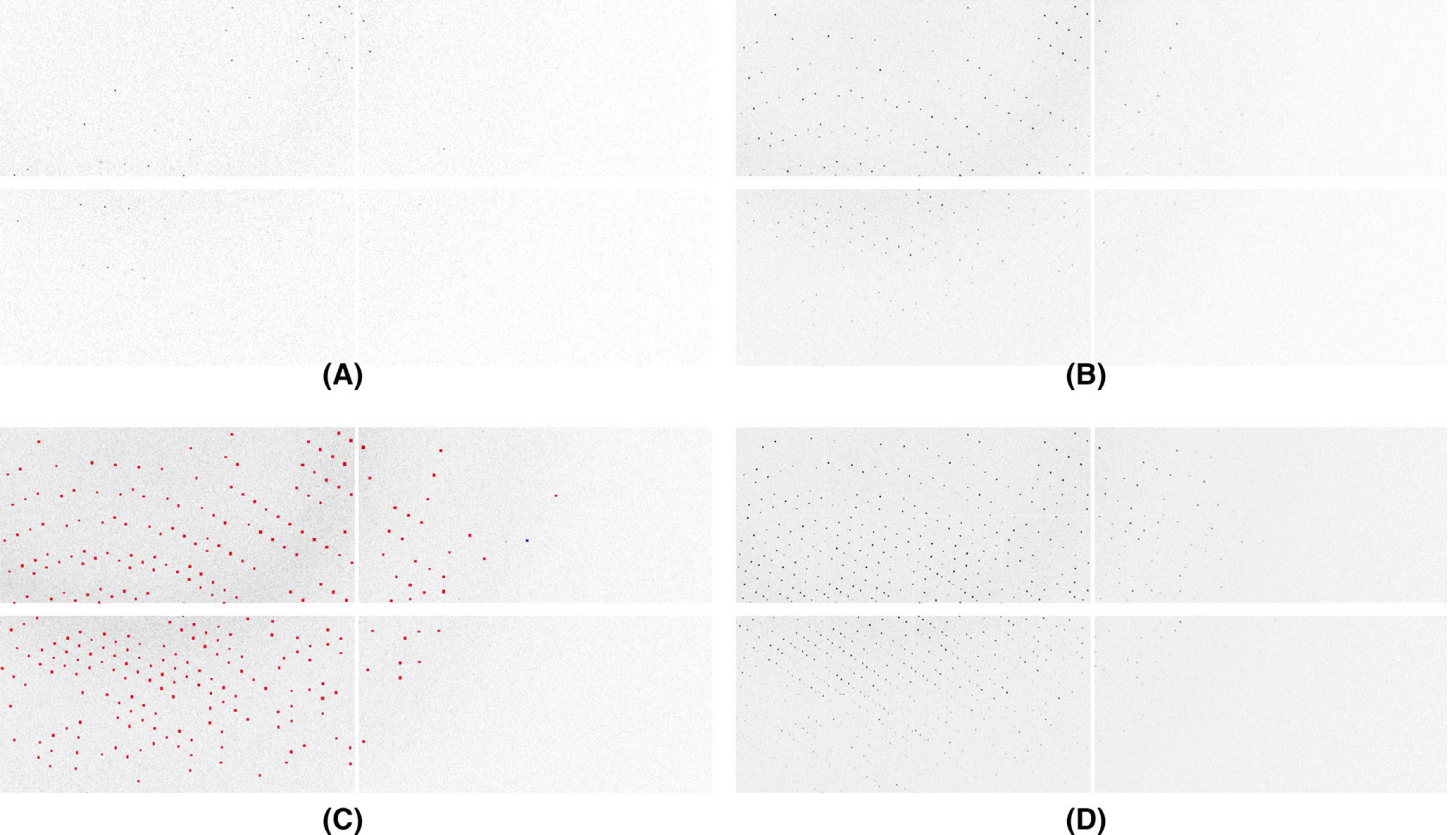
Zoomed‐in views of four modules of an EIGER 2XE: (a) a single image, (b) the maximum pixels from a 10 image “stack” (corresponding to an image width of 1°), (c) the same with the “shoeboxes” from spot finding superimposed and (d) the maximum value of pixels in a 25 image stack

Even this simple workflow offers an opportunity to demonstrate a number of useful DIALS tools for the inspection of data: the image viewer and reciprocal lattice viewer. The image viewer allows the processing results to be superimposed onto the detector images, as well as showing stacked images (Figure 4), which gives the user an opportunity to more easily inspect finely sliced pixel‐array detector data.

#### 3.1.1 General usage

Every DIALS program provides a very brief summary of its function when run with the ‐‐help command line parameter.

DIALS programs generally interact with three kinds of files:

- image files, such as .h5 or .cbf,
- DIALS reflection files (.refl) which contain the reflection data, and
- DIALS experiment files (.expt) which contain experimental models.

The command‐line parameters are provided in PHIL format^4^ which takes the form option = value where option can be nested. The available options for any DIALS program can be found by running






  dials.program -c -e1






For example, spot finding can be restricted to looking only within a specific resolution range by specifying d_min = 2.0 d_max = 40.

#### 3.1.2 Import

The first stage of data processing with DIALS is to import the data: this does not actually read the pixel values, but reads the image headers and associated metadata to build a model of the experiment. It is at this stage that any prior knowledge of the experimental set‐up is best applied, for example, overriding the beam center. In most cases, the authors would hope the experimental parameters are correctly recorded so all that is necessary is






  dials.import /path/to/image_*.cbf






for data from a DECTRIS PILATUS detector, or






  dials.import /path/to/image_master.h5






for data from a DECTRIS EIGER detector. If you only wish to import a subset of the images in an EIGER data set then image_range = start,end may be used, which will work with images from start to end inclusive. In most cases the parameters fast_slow_beam_centre and distance should be sufficient to correct any misrecording of the metadata.

#### 3.1.3 Find spots

The first “real work” of processing diffraction data with DIALS is to perform spot finding. In contrast to MOSFLM and HKL3000, the spot finding in DIALS is performed on all the images to be analyzed. While this is computationally expensive, it does allow a more complete model of the data to be constructed before integration begins. In general the defaults are sensible, and the authors rarely find themselves adjusting the parameters. As illustrated above, however, there are a number of parameters which can be tuned to optimize the spot finding in more challenging situations.

**FIGURE 5:**
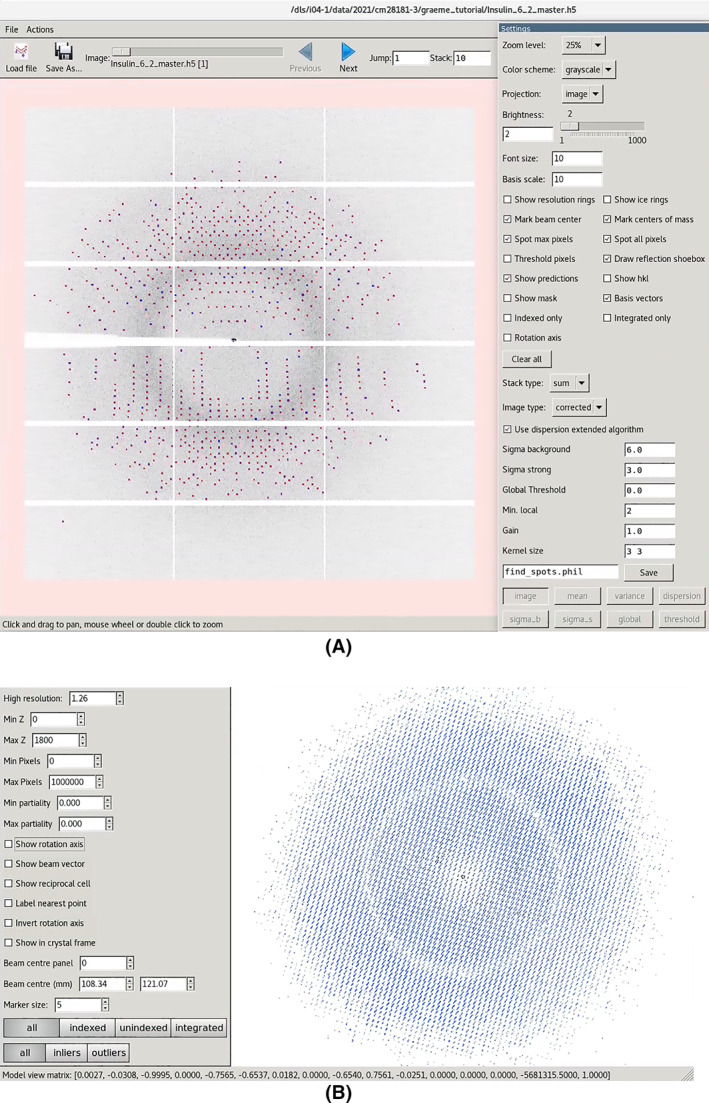
DIALS image viewer (top) and reciprocal lattice viewer (bottom), showing the outcome of spot finding on a data set from a crystal of cubic insulin, recorded on a DECTRIS EIGER 2XE 9M detector. The image viewer has 10 images stacked, giving 2° of rotation, while the reciprocal lattice view is the entire data set

After spot finding, the output file (strong.refl, by default) contains the bounding boxes, centroids, and pixels of all the features found. These may be visualized either by superimposing them onto the diffraction images (Figure 5 upper) with dials.image_viewer imported.expt strong.refl or in reciprocal space with dials.reciprocal_lattice_viewer imported.expt strong.refl (Figure 5 lower). These tools are invaluable for diagnosing issues with the metadata as well as inspecting the processing in later DIALS analysis steps.

#### 3.1.4 Indexing and refinement

The next stage in processing is indexing and refinement. In this context, “indexing” includes basis determination and can optionally include an evaluation of the correct Bravais lattice to use. All indexing procedures follow a similar protocol:

- map points to reciprocal space using the current experimental geometry model,
- seek periodicity in the reciprocal space positions with a one‐ or three‐dimensional fast Fourier transform, or other algorithms,
- find a set of three basis vectors which span a maximal set of reciprocal lattice positions,
- assign indices with this basis,
- refine lattice parameters and geometry using the mapping between observed and predicted locations.

In dials.index the main choice is the basis vector search algorithm to use, that is, one‐ or three‐dimensional fast Fourier transform, or real space grid search,^22^ and optionally the unit cell or space group. For most rotation data sets, the default settings (unknown unit cell and symmetry, 3D FFT) are robust. The indexing process is iterative, first indexing reflections to a relatively low resolution, then refining the model and geometry, before using the updated models to index higher resolution reflections. At the end, an indication of the overall fraction of reflections indexed is reported: if this is significantly below 50% there may be multiple lattices present in the data.

Multiple independent lattices may be indexed by using the max_lattices option, for example, setting to 2 if around half of the reflections were indexed.

After the initial indexing and refinement, an optional step is to assess possible Bravais lattices. This takes the “shape” of the primitive basis and attempts to estimate all possible lattices using code derived from iotbx.lattice_symmetry.^23^ For each lattice, the data are then reindexed and some simple refinement is performed: the quality of the fit for each lattice can then be assessed by considering the impact on the root mean square deviations and the Le Page *δ* parameter.^24^ Each solution is output as a reindexed experiment file, however the data are not copied, so before proceeding to refinement the reflections need to be reindexed using the selected solution. It is worth noting, however, that it is perfectly valid to perform processing with a triclinic lattice, then assign the crystal symmetry prior to scaling.

**FIGURE 6:**
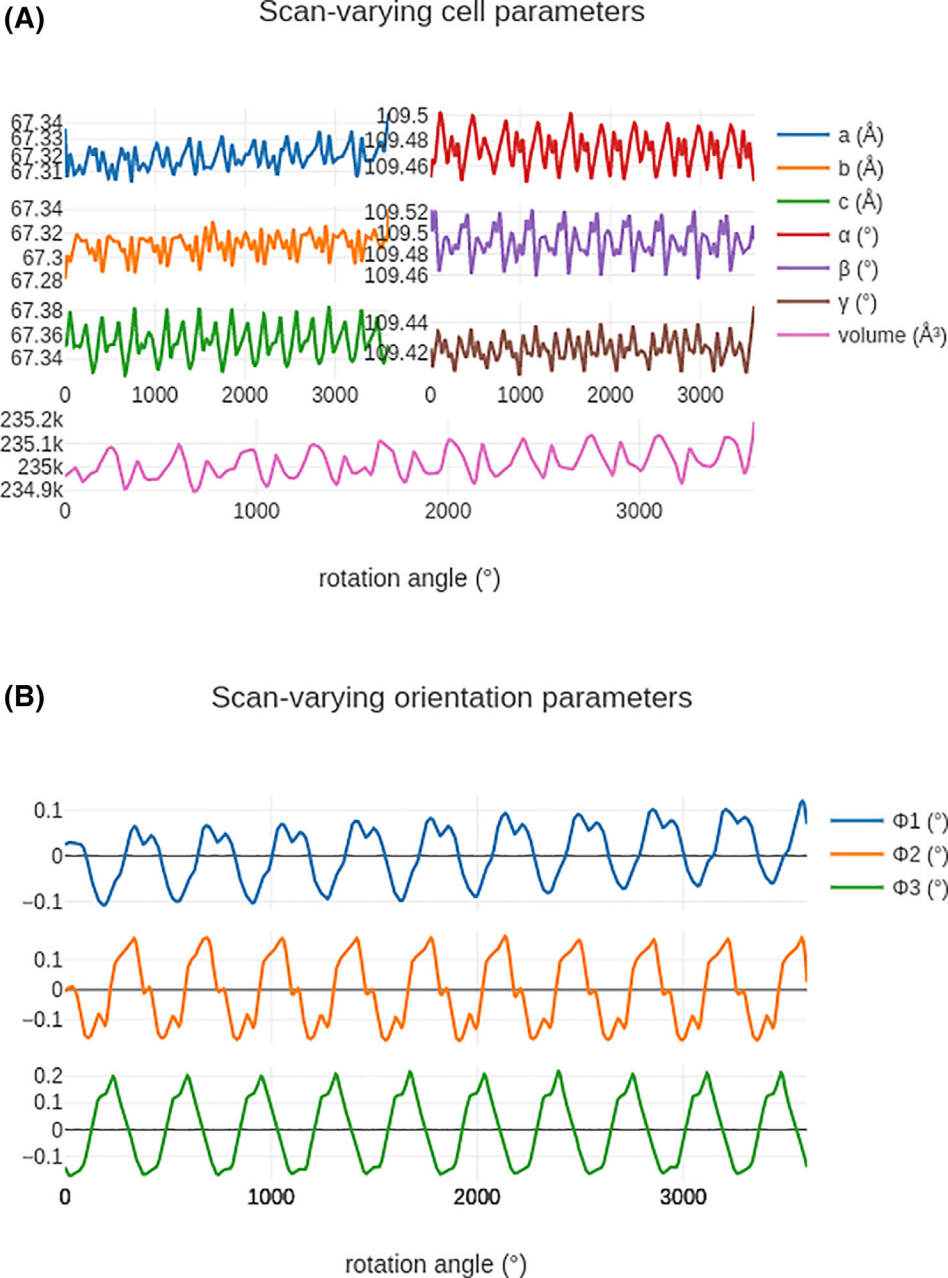
Scan‐varying cell parameters (top) and orientation (bottom) of cubic insulin rotated through 3,600°. The best‐fit unit cell parameters clearly vary depending on the observed angle. This could be due to variations in crystal uniformity as it rotates through the beam

After indexing or reindexing, the models should be further refined to more accurately predict the observations. By default, the refinement will first be performed with a static model, before allowing for a scan varying model, in which the unit cell and crystal orientation are allowed to vary smoothly over the course of the rotation scan.^25^ In the absence of radiation damage, it may be expected that the unit cell of the crystal remains constant during the experiment. In practice, if the crystal is not entirely bathed in the X‐ray beam, or the beam does not have a perfectly uniform flux density, the weighted average unit cell may vary slightly as the sample rotates, due to variations in the unit cell across the sample. This is illustrated in Figure 6, which shows analysis of a substantial cubic insulin data set recorded on Diamond Light Source beamline I04‐1.^5^ The scan varying unit cell dimensions, angles and sample orientation are shown as the crystal is rotated through 3,600°: the variations improve the overall alignment between the predictions and reflection observations, but care should be taken in the physical interpretation, as the overall cell volume may depend on very small changes in the effective changes in the detector distance as the sample rotates. The scan varying refinement does however substantially improve the prediction of the diffraction pattern, in this example reducing the RMS deviations by a factor of two in the detector plane and over three in rotation thus ensuring that the profile parameters for the subsequent integration step are more appropriate.

#### 3.1.5 Integration

While integration is one of the most demanding data processing steps, there are very few parameters to adjust, as most of the information needed is derived from earlier stages. The main choice is whether to use profile fitting or simple summation integration. DIALS uses profile fitting by default, as this can give more accurate measurements of weak reflections.^1^ To use simple summation integration you can provide the option profile.fitting = false on the command line. After the data have been integrated, it is possible to view the reflection shoeboxes in the image viewer, discussed below.

The flow of integration is to first inspect the indexed reflection profiles of the reflection shoeboxes saved during spot finding: this determines two *σ* parameters^9^ defining the reflection extent. *σ*_*m*_ describes the rotational direction (e.g., mosaicity) while *σ*_*b*_ describes the size on the detector face—these simply define the extent of the profile model volume, such that the transformed profiles should correspond to approximately the central one third of the model in every direction and do not imply a Gaussian shape.

If profile fitting, the next stage is to gather reference profiles from the data set by predicting the spot locations and transforming the density from strong reflections to reciprocal space. These reference profiles are then used to model all reflections in a second pass. For summation integration the region defined by the *σ* parameters is assumed to contain the reflection peak with the surrounding region background.

**FIGURE 7:**
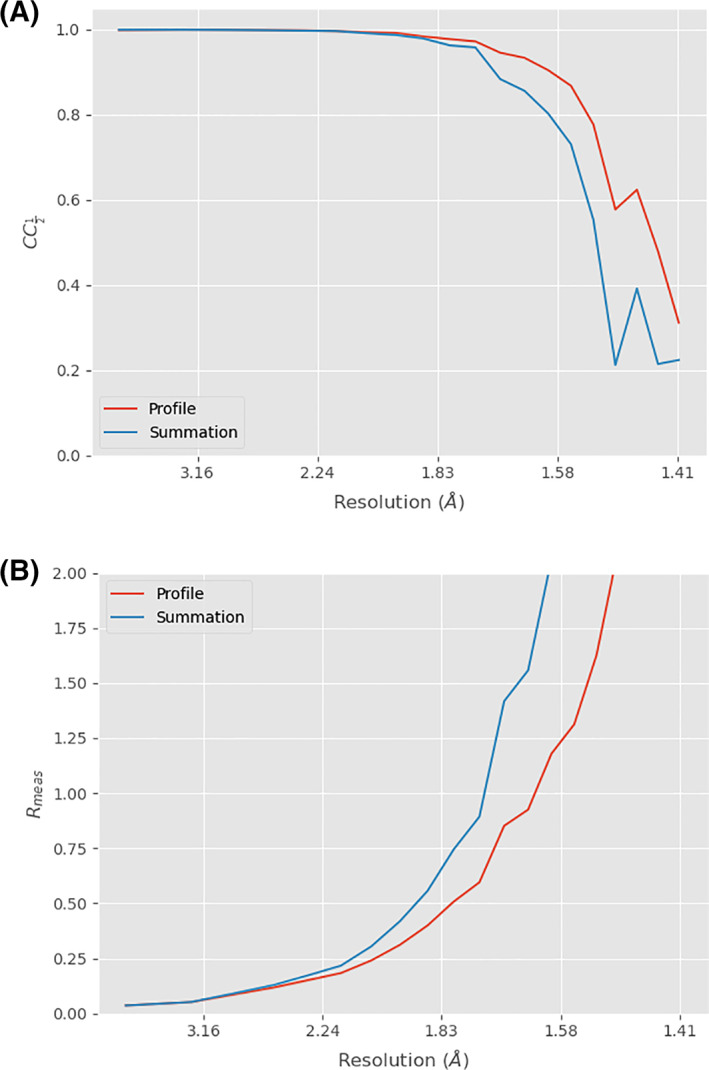
CC½ (top) and *R*_meas_ (bottom) for both profile‐fitted and summation integrated cubic insulin data. The data from profile fitting have both a superior half set correlation and lower merging residual at high resolution, where the reflections are at their weakest. At low resolution the figures are similar for both methods

In the absence of background, the results from summation integration and profile fitted integration should be similar. In most cases, however, there is some background scatter, and profile fitting helps to give a more reliable measurement of weak reflections. This is best illustrated by comparing the merging statistics from processing a “typical” data set with and without profile fitting: in this case a smaller cubic insulin data set recorded in the same session as the data above. The data were processed following the basic DIALS workflow described earlier, with and without profile fitting, then comparing the scaled but unmerged measurements For this purpose we use the program xia2.compare_merging_statistics, described in the Appendix. The results are shown in Figure 7: the strong reflections at low resolution agree very well in both cases, but the weaker reflections have lower residuals (i.e., better agreeing measurements) when using profile fitting.

#### 3.1.6 Symmetry determination and scaling

Even where a lattice was assigned earlier, at the indexing stage, it is necessary to assign the correct symmetry to the data before scaling. dials.symmetry can determine this automatically by assessing the highest possible symmetry compatible with the unit cell, then testing every possible subgroup of this symmetry, following the methods pioneered in POINTLESS.^10^ A noteworthy feature of DIALS, however, is that the data can first be scaled in P1 before symmetry determination. In the case of significant diffraction anisotropy, this may improve the correlation in the symmetry operators and thus the “signal” on which the decision of the correct space group is determined.

In scaling the data, there are a handful of options to consider, though the defaults are appropriate in the majority of cases. The principal choice is whether to adopt a “physical” model for the scaling correction (following the approach taken in AIMLESS,^11^ modeling the sample scale, decay and absorption as smoothly‐varying physical processes) or using an “array” model (similar to that in XDS) which has rather more parameters but could deal more gracefully with discontinuities in the data. In either case, the option anomalous = true can be set, which will cause the program to treat *I*_*hkl*_^+^ and *I*_*hkl*_^−^ as independent observations. In the case of the default physical absorption correction model the option absorption_level = low, medium or high can be used to give the program an indication of the extent to which to constrain the relative absorption correction, which may be significant with longer wavelengths or inorganic samples.

The output from scaling includes a variety of metrics useful for assessing data quality. In addition to overall and resolution‐binned merging statistics, a smooth curve‐fitting of CC½ versus resolution is used to indicate the resolution limit at which CC½ = 0.3. The user is also encouraged to inspect the “error model” used during scaling to adjust *σ*(*I*), which can indicate the extent of systematic errors in the dataset. Depending on the data quality it may be desirable to trim and rescale the data. For example, where a range of images, say 101 up to 120, have bad R‐merge values (which can be inspected in the HTML report output by scaling), they can be excluded by rescaling the scaled output files with the option exclude_images = 101:120.

#### 3.1.7 Postintegration cell refinement

Depending on the flow chosen through the processing, it is possible at the point of scaling that the unit cell may never have been refined against the data with the symmetry constraints applied, though in most cases this is unlikely to have a significant impact as the unit cell parameters are significantly overdetermined. Particularly in chemical crystallography the uncertainties in the unit cell may however be of interest in subsequent analysis. DIALS features a tool dials.two_theta_refine that minimizes the unit cell constants against the difference between observed and calculated 2*θ* values, which are determined from background‐subtracted integrated centroids. This can be used to derive a unit cell that is a suitable representative average in subsequent processing. For example, we can compare the result of refinement of the cell against scaled data:






Final refined crystal model:



Crystal:



 Unit cell: 57.75947(4), 57.75947(4), 149.88010(14), 90.0, 90.0, 90.0



 Space group: P 41 21 2






with the unit cell from the refinement prior to integration:






Final refined crystal model:



Crystal:



 Unit cell: 57.75886(16), 57.76935(16), 149.8966(4), 90.01671(5), 89.99836(5), 90.00664(6)



 Space group: P 1






It may be seen that the cell parameters are very slightly different, due to being derived from properly background‐subtracted measurements, and have smaller uncertainties. This method has the advantage of being independent of variation in orientation of the sample, though of course it may not be appropriate if the sample has undergone significant radiation damage.

This method is particularly useful for determining an overall “best” unit cell for multi‐crystal data, and is used as part of xia2.multiplex.

#### 3.1.8 Exporting data

The processing described above has used DIALS file formats for the reflection data and experiment models. These are, however, not supported in downstream analysis, so the final step is to export the measurements in a suitable form for use by other software. The default is the CCP4 MTZ format, accessed as:






  dials.export scaled.refl scaled.expt






which will export the scaled but unmerged data to MTZ format. This may be re‐merged with tools like AIMLESS when imported into CCP4. Alternatively, the data may be merged in DIALS before output, using dials.merge, which includes an implementation of the French & Wilson algorithm^26^ to compute structure factors from the merged intensities.

#### 3.1.9 Generating a report

**FIGURE 8:**
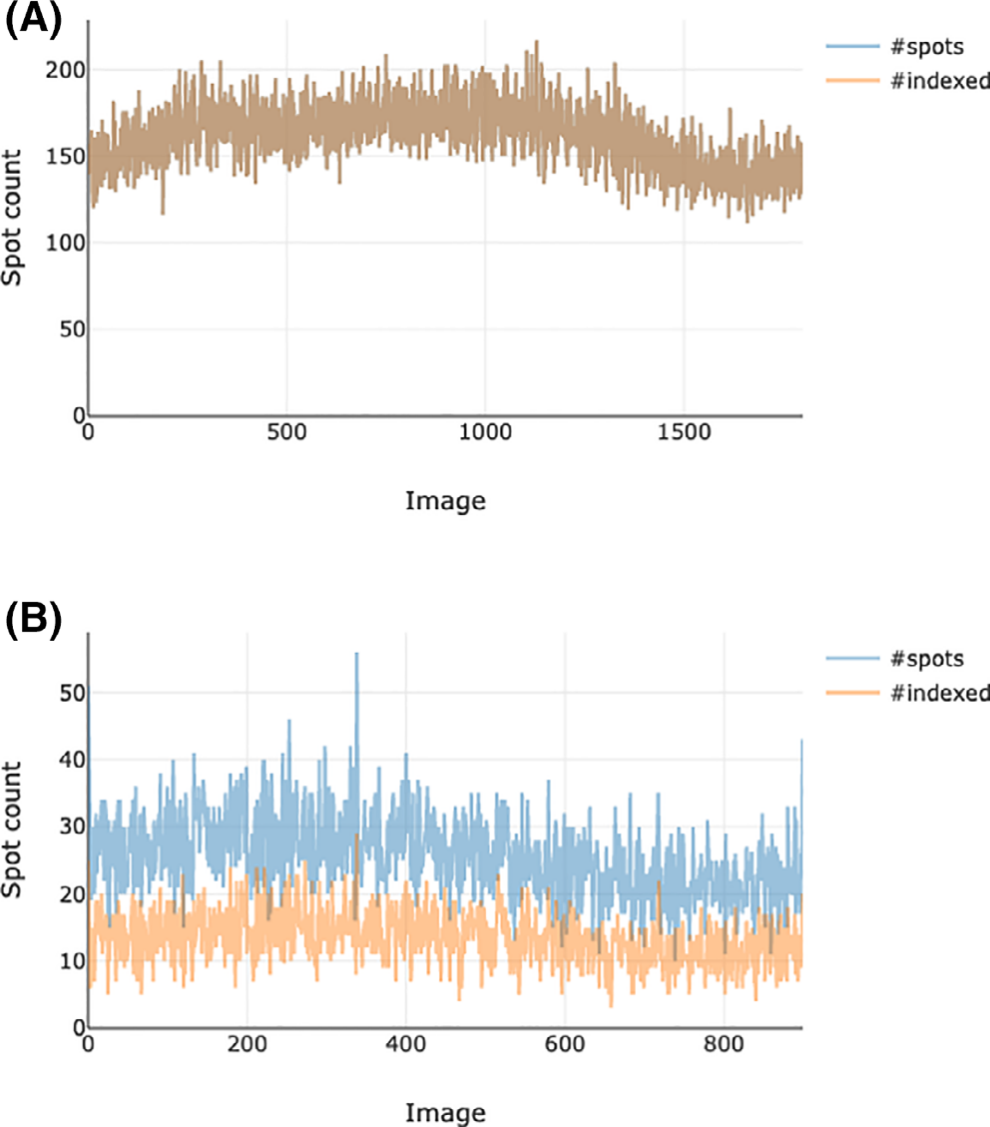
Fraction of reflections indexed in a macromolecular case with a strong single lattice (top), and a small molecule data set with two distinct lattices (bottom). In the upper image, it is clear that the vast majority of reflections are indexed. In the lower, the fact that about half are indexed suggests that there is a second (unindexed) lattice in the data set

At any stage in processing with DIALS, the output may be used to generate a HTML report using dials.report. These are incremental, such that the report of a given step includes the analysis derived from all previous steps. This provides an opportunity to view a visual representation of much of the analysis, for example, the fraction of indexed reflections (Figure 8).

### 3.2 Viewers

While the primary focus of DIALS development was very much on a suite of command‐line tools to enable automated data processing, some graphical tools have been developed, in part to enable DIALS development but also to provide alternative methods of visual feedback. The most useful are dials.image_viewer and dials.reciprocal_lattice_viewer. The former, as the name suggests, allows one to view the image data, but more importantly also allows an overlay of the current state of processing: found spots, indexed spots, and integration shoeboxes can be drawn over the images, which may also be stacked.

**FIGURE 9:**
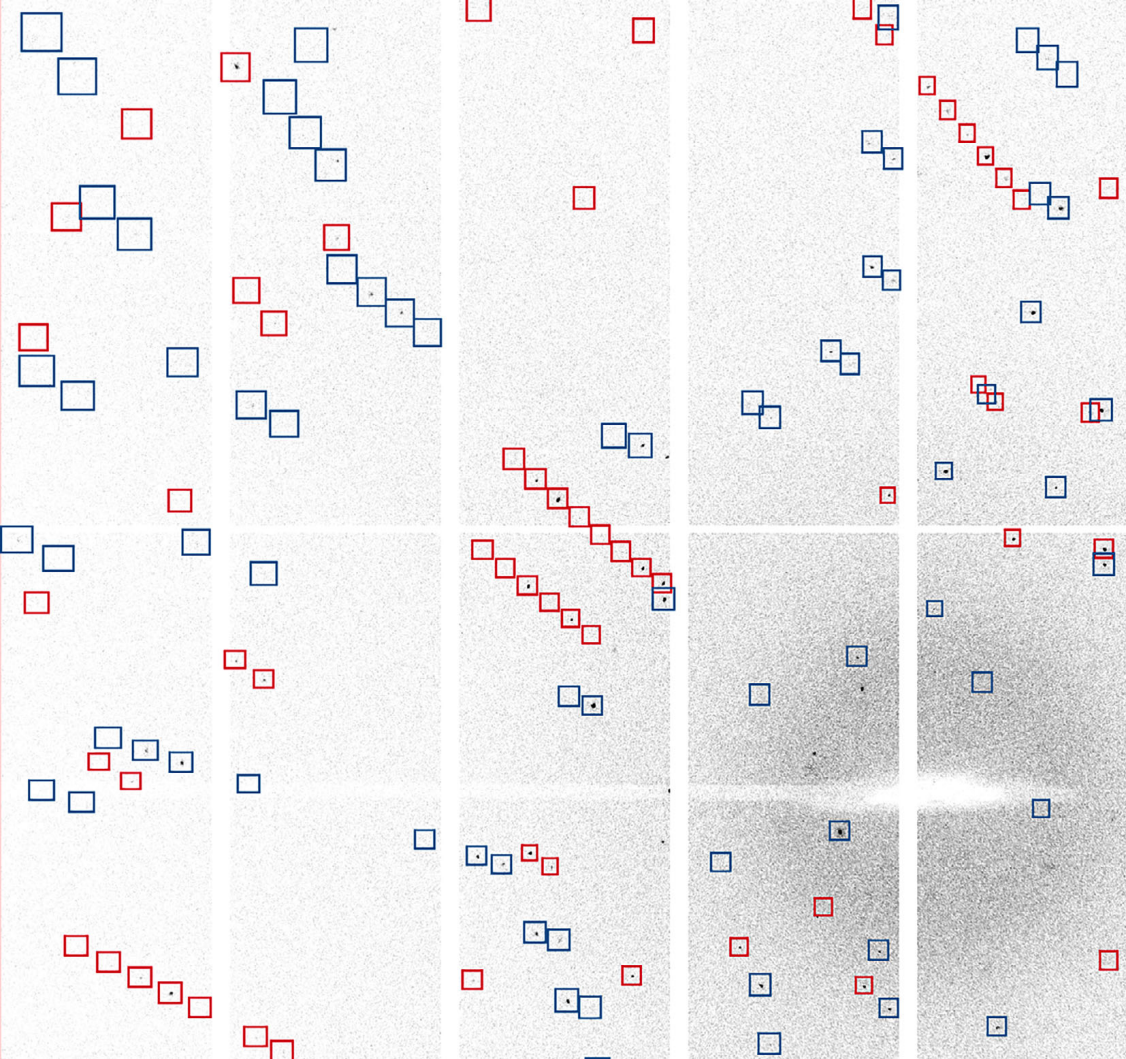
View captured from the image viewer showing a zoomed‐in view of reflection shoeboxes from integrating a two‐lattice small molecule data set. The boxes correspond to the entire reflection, including background with the red and blue boxes represent different lattices. The detail of which *pixels* are peak and background are not saved by default

Figure 9 shows a zoomed view of the integration shoeboxes from the multi‐lattice data set mentioned above, showing that the bounding boxes are largely well spaced. Stacking these images (not shown) quickly leads to overlapping shoeboxes demonstrating the value in using narrow oscillations.

**FIGURE 10:**
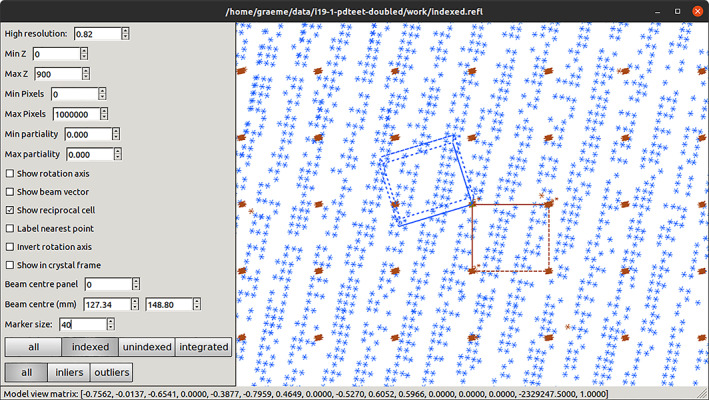
Reciprocal lattice view of a data set with two lattices present, showing the relative orientation of the reciprocal cells

In contrast to the image viewer, the reciprocal lattice viewer gives a sense of the overall properties of the data set. Figure 10 shows the reciprocal lattice view of the same data, with one lattice aligned: here it is clear that the two lattices have different orientations.

## 4 ADVANCED COMMAND‐LINE TOOLS

The tools shown thus far have emphasized the processing of data from a single crystal. In some cases it is impossible to record a complete data set from a single sample, and data are recorded from multiple samples. As mentioned earlier, xia2.multiplex is available as a high‐level tool. By design this performs a number of different steps (e.g., resolution of any indexing ambiguity, as well as scaling and isomorphism analysis). Within DIALS the lower‐level tool dials.cosym ^15^ is available to resolve the alignment of data from multiple samples in reciprocal space, which may be used to implement a beamline feature for real‐time feedback for multi‐crystal data collection.

### 4.1 Isomorphism analysis: dials.cosym

The intention of dials.cosym was simple: to allow resolution of indexing ambiguity when the crystal symmetry was unknown. The Brehm‐Diederichs algorithm^27^ was a novel approach to resolving indexing ambiguity when the true space group was known to be polar. dials.cosym extends this to consider the problem of crystal symmetry and indexing ambiguity simultaneously, by assuming that the data are from a triclinic crystal then testing every possible *lattice* symmetry operator as a possible twinning operator. In practice, dials.cosym will rapidly identify those lattice symmetry operators present in the data and the true twinning operations, and aligns the data sets in a consistent manner in reciprocal space. Subsequently, dials.symmetry can be used to identify the true symmetry of the data followed by scaling with dials.scale.

### 4.2 *Isomorphism analysis: ΔCC* ½

Following the use of dials.cosym to identify and eliminate indexing nonisomorphism, the isomorphism of scaled intensities can be assessed using ΔCC*½* values as described in.^28^ Within DIALS, this algorithm is implemented in the tool dials.compute_delta_cchalf. In this approach, a ΔCC½ value for a subset of the data is determined by calculating the difference between the full‐dataset CC½ value and the value excluding that subset. A negative ΔCC½ for a particular subset of the data indicates that the overall intensity correlation is improved by removing that subset, which provides a quick means of feedback as to the most nonisomorphous subsets of the data. The toolkit nature of DIALS means that features such as the ΔCC½ analysis can easily be combined with other tools to construct processing workflows. For example, within dials.scale a feature was implemented to allow successive cycles of scaling and ΔCC½ analysis, to iteratively improve the data until a convergence criterion is reached, as discussed in Reference 12.

## 5 PYTHON API

DIALS was implemented using a “hybrid” programming method in which code is written as a mix of Python and C++. The former is very flexible and easy to work with, while the latter is better for high performance calculations. In general, the pattern is that the “glue” code (interaction with user, etc.) is written in Python and the low level algorithms in C++, which makes it possible to apply those algorithms in novel situations by simply writing a short Python script.

Two examples follow:

- Identification of saturated detector pixels
- Detection of bad / misbehaving pixels in a pixel array detector

The intent behind these examples is to provide the reader with a primer on how to access algorithms in Python and makes no effort to be comprehensive.

### 5.1 Detecting overloaded pixels

With pixel array detectors such as the DECTRIS EIGER and PILATUS it can be possible to have “saturated” pixels, that is, pixels where the total counts exceed an allowed amount, which depends on settings such as the gain and exposure time. screen19, described above, is intended to identify such cases, however as a user you may be interested in exactly where these overloads occurred. In DIALS spot finding it is possible to override the upper range of the trusted pixel range, from, for example, 4,231 for very short exposure times to 65,536. Spot‐finding would then retain those pixels above the true trusted range allowing overloaded spots to be found. By default, the spot finding in DIALS makes a copy in the output file of the cuboid surrounding the spot, so it becomes straightforward to scan for saturation among the pixels belonging to the found spots.

When running dials.find_spots it is necessary to assign a higher limit to maximum_trusted_value, after which spot finding will proceed normally. The strong reflection file may then be analyzed with:






import sys



from dials.array_family import flex



data = flex.reflection_table.from_file(sys.argv[1])



boxes = data["shoebox"]



nn = boxes.size()



h0 = flex.histogram(flex.double(), data_min=0, data_max=5000, n_slots=5000)



for j in range(nn):



    h1 = flex.histogram(boxes[j].data.as_double().as_1d(), data_min=0, data_max=5000, n_slots=5000)



**FIGURE 11:**
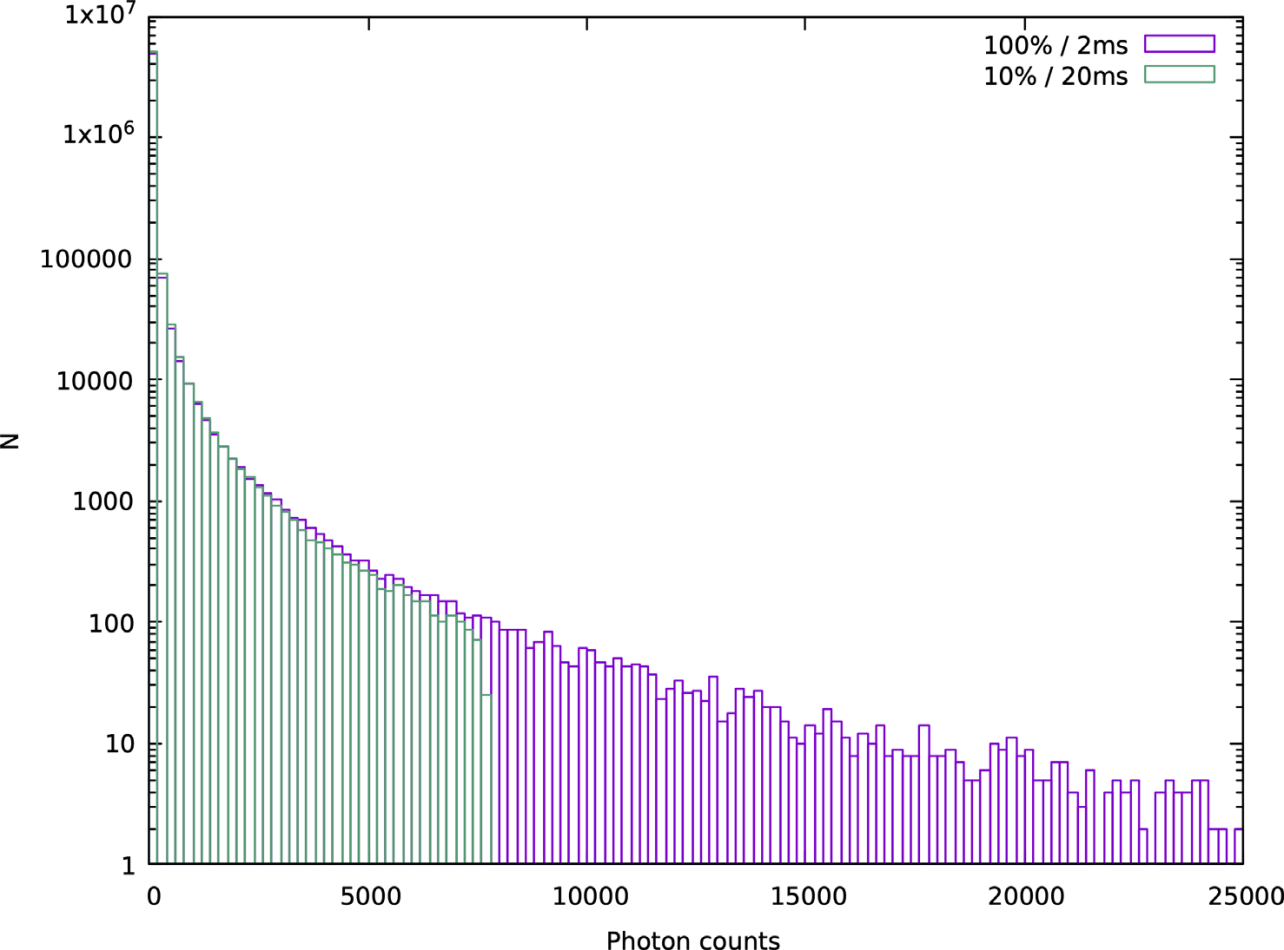
Pixel intensities drawn from strong pixels from two data sets collected in an apparently equivalent manner, though the faster collection clearly saturates the detector

    h0.update(h1)



for c, v in zip(h0.slot_centers(), h0.slots()):



    print(c, v)






which builds up a histogram of all the pixels in the strong spots. From this, it is clear to see that, while there are pixels above the trusted range, the distribution is clearly truncated and does not match the expected exponential distribution (Figure 11).

### 5.2 Detecting bad pixels

The arrival of pixel array detectors revolutionized the collection of X‐ray diffraction data. The ability to read out all pixels with essentially zero read‐out noise and read‐out time enabled continuous, shutterless rotation data collection. Since every pixel has its own read‐out electronics, it is possible for some pixels to fail, either reading out nothing or otherwise giving unreliable values. To complicate matters, it can be the case that pixels may “twinkle,” reading out zero or some arbitrary value on alternate reads if the detector has double readout, as with the DECTRIS EIGER 2 XE.

Detecting these pixels could be as simple as reading out with no X‐ray exposure and then thresholding. This would, however, miss pixels which only misread when some charge is accumulated. A more robust method, implemented below, is to use the algorithms behind spot finding to identify pixels with signal and then accumulate the number of times each pixel contains signal. If this is the case for a half or more of the images, it is unlikely that the pixel is reliable. Such a method has the advantage that it can be used with routinely collected X‐ray diffraction data, to give a more reliable test.






detector = imageset.get_detector()[0]



trusted = detector.get_trusted_range()



total = None



for idx in range(100):



    pixels = imageset.get_raw_data(idx - 1)



    known_mask = imageset.get_mask(idx - 1)



    # apply known mask



    for pixel, panel, mask in zip(pixels, panels, known_mask):



        pixel.set_selected(~mask, -1)



        for f0, s0, f1, s1 in panel.get_mask():



            blank = flex.int(flex.grid(s1 - s0, f1 - f0), 0)



            pixel.matrix_paste_block_in_place(blank, s0, f0)



        data = pixels[0]



        negative = data < int(round(trusted[0]))



        hot = data > int(round(trusted[1]))



        bad = negative | hot



        data = data.as_double()



        p = spot_phil.fetch(



        source=iotbx.phil.parse("min_spot_size=1")



        ).extract()



        threshold_function = SpotFinderFactory.configure_threshold(p)



        peak_pixels = threshold_function.compute_threshold(data, ~bad)



        if total is None:



        total = peak_pixels.as_1d().as_int()



        else:



        total += peak_pixels.as_1d().as_int()






## 6 EXTENSION TO OTHER TECHNIQUES

So far, we have showcased the modular properties of DIALS by showing how easy it is to update the individual algorithms and their parameters for various applications in X‐ray rotational crystallography. The established toolkit of algorithms that make up DIALS can be expanded to different experimental geometries (see Reference 29 for DIALS applied to X‐ray serial crystallography) and, more generally, to different diffracting particles. DIALS is being actively developed to accommodate the needs of these other diffraction communities just as their user bases are expanding.

In recent years, electron diffraction has emerged as a tool that is capable of determining structures from nanocrystals, from small molecules through to biological macromolecules,^30^ using a method usually termed MicroED or 3DED. The data collection geometry employed for this method has gradually converged with the standard rotation method used in X‐ray crystallography, resulting in a relatively accessible tool that can resolve structures of crystals once considered too small to study, thus complementing X‐ray crystallography methods. This also implies that data analysis software used for X‐ray crystallography could be extended to electron diffraction without significant disruption to the standard workflow. For DIALS to successfully process continuous rotation electron diffraction data,^31^ slight adaptations were required at various steps to account for incongruities in experimental set up and Ewald wedge size covered. Similar approaches to extending DIALS to further electron diffraction methods, such as serial ED, are also underway.

Additionally, DIALS is also being extended to neutron single crystal diffraction. In this case, an initial focus is being given to time‐of‐flight (ToF) Laue data from the SXD beamline at ISIS.^32^ This is a significant departure from monochromatic X‐rays, where each reflection must be processed with respect to its ToF and hence wavelength. Nevertheless, the modular nature of the DIALS workflow in Figure 3 means the process can be readily adapted, interfacing to neutron‐specific algorithms where appropriate. This includes adapting the DIALS image viewer to work with 3D ToF data, where users can view harmonics at a given panel location, additional ToF residual terms during refinement, additional integration algorithms in reciprocal space to better account for spallation source peak profiles, and more comprehensive corrections during scaling, such as modeling the sample volume. This work is set to be in production during the second quarter of 2022, with plans to extend to monochromatic and Quasi‐Laue neutron sources, as well as the proposed LMX and NMX beamlines at ISIS and ESS, respectively.

The approach of treating DIALS as a toolkit is being extended to these new methods, such that they are designed to be used both within DIALS and from other packages. For neutron diffraction data, initial work has focused on allowing users to easily convert from DIALS formats to those in complimentary packages like Mantid,^33^ such that algorithms from both can be utilized at different stages of the processing workflow.

## 7 DISCUSSION AND CONCLUSIONS

We have described how the DIALS package functions as a general‐purpose toolbox for crystallographic data processing. The tools allow various degrees of convenience and customization. At the highest level, they perform the routine processing of single‐crystal diffraction data from a range of sample types and techniques, in a manner similar to other established data processing packages. However, unlike those packages, the user is also able to delve deeper, developing new tools to answer specific questions or add new capabilities to the software. The choice to develop the software in an open manner allows others to report issues, inspect and even contribute to the code, at https://github.com/dials/dials. As with most software, DIALS is not “finished” and there is scope for further development, however the package has been used in a substantial number of PDB depositions (some 3,500 or so, at the time of writing) as well as facilitating automated data processing at Diamond Light Source and elsewhere.

This description is not exhaustive, simply aiming to give the reader a flavor of what is available in the package rather than a comprehensive atlas of its capabilities. More detailed documentation is available at https://dials.github.io, which includes tutorials on how to apply the DIALS command line tools to for example, chemical crystallography and electron diffraction data.

Finally, the software is openly licensed (BSD) with the intent that third parties may distribute and use the code however they see fit—this choice was made deliberately to maximize the return on the grant and public funding we have received over the last decade.

## AUTHOR CONTRIBUTIONS

**Graeme Winter:** Conceptualization (equal); funding acquisition (equal); investigation (equal); methodology (equal); software (equal); supervision (equal); writing – original draft (lead); writing – review and editing (equal). **James Beilsten‐Edmands:** Conceptualization (equal); investigation (equal); methodology (equal); software (equal); visualization (equal); writing – original draft (supporting); writing – review and editing (supporting). **Nicholas Devenish:** Conceptualization (equal); investigation (equal); methodology (equal); software (equal); visualization (equal); writing – original draft (supporting); writing – review and editing (lead). **Markus Gerstel:** Conceptualization (equal); investigation (equal); methodology (equal); software (equal); visualization (equal); writing – original draft (supporting); writing – review and editing (supporting). **Richard J Gildea:** Conceptualization (equal); investigation (equal); methodology (equal); software (equal); visualization (equal); writing – original draft (supporting); writing – review and editing (supporting). **David McDonagh:** Conceptualization (equal); investigation (equal); methodology (equal); software (equal); visualization (supporting); writing – original draft (supporting); writing – review and editing (supporting). **Elena Pascal:** Conceptualization (equal); investigation (equal); methodology (equal); software (equal); visualization (equal); writing – original draft (supporting); writing – review and editing (supporting). **David G Waterman:** Conceptualization (equal); funding acquisition (equal); investigation (equal); methodology (equal); software (equal); supervision (equal); visualization (equal); writing – original draft (supporting); writing – review and editing (supporting). **Benjamin H Williams:** Conceptualization (equal); investigation (equal); methodology (equal); software (equal); visualization (equal); writing – original draft (supporting); writing – review and editing (supporting). **Gwyndaf Evans:** Conceptualization (equal); funding acquisition (lead); investigation (equal); methodology (equal); supervision (equal).

## Endnotes

^1^

https://plotly.com

^2^

https://github.com/xia2/screen19

^3^
A script illustrating this is included in the Appendix.

^4^

https://cci.lbl.gov/cctbx_docs/libtbx/libtbx.phil.html

^5^

https://doi.org/10.5281/zenodo.5155653

## Commands used in manuscript preparation

### A short DIALS command script

The simple workflow of DIALS may be executed with the following commands:

dials.import ${DATA}

 dials.find_spots imported.expt

 dials.index imported.expt strong.refl

 dials.refine indexed.expt indexed.refl

 dials.integrate refined.expt refined.refl

 dials.symmetry integrated.expt integrated.refl

 dials.scale symmetrized.expt symmetrized.refl anomalous=true

 dials.export scaled.expt scaled.refl

 dials.merge scaled.expt scaled.refl

Provided the content of the image headers is reasonably accurate, in the majority of cases this will give usable data for subsequent analysis steps, though the resolution limit will not have been constrained and no effort made to reduce the extent of the data in case of radiation damage.

### Comparing scaled data

The comparison between summation integrated and profile fitted integration was made using the xia2.compare_merging_statistics tool, which reads scaled but unmerged data in MTZ format, and plots the merging statistics on adjacent figures. While this is used here to compare data from two different methods of DIALS processing, it is entirely possible to perform the same comparison between for example, data processed with XDS and DIALS.

xia2.compare_merging_stats profile/scaled.mtz sum/scaled.mtz \

small_multiples=1 plot_labels="Profile Summation" d_min=1.4

Here the small_multiples option shows all curves on all plots as a pale gray, with the curve of interest in red to simplify comparison.
